# *N*′-(3-Bromo-5-chloro-2-hydroxy­benzyl­idene)-4-hydr­oxybenzohydrazide

**DOI:** 10.1107/S160053680802905X

**Published:** 2008-09-13

**Authors:** Ling-Wei Xue, Yong-Jun Han, Cheng-Jun Hao, Gan-Qing Zhao, Qiao-Ru Liu

**Affiliations:** a College of Chemistry and Chemical Engineering, Pingdingshan University, Pingdingshan Henan 467000, People’s Republic of China

## Abstract

The mol­ecule of the title compound, C_14_H_10_BrClN_2_O_3_, is planar [dihedral angle between the aromatic rings = 3.0 (2)°] and shows a *trans* configuration with respect to the C=N double bond. The crystal structure is stabilized by inter­molecular N—H⋯O hydrogen bonds and an intramolecular O—H⋯N interaction also occurs.

## Related literature

For the biological properties of Schiff bases, see: Bhandari *et al.* (2008 ▶); Sinha *et al.* (2008 ▶); Sondhi *et al.* (2006 ▶); Singh *et al.* (2006 ▶). For background on Schiff bases derived from aldehydes with benzohydrazides, see: He & Liu (2005 ▶); Zhen & Han (2005 ▶); Diao & Yu (2006 ▶); Shan *et al.* (2008 ▶); Fun *et al.* (2008 ▶). For related structures, see: Jing *et al.* (2005 ▶); Lu *et al.* (2008 ▶); Salhin *et al.* (2007 ▶).
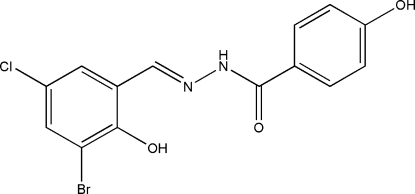


## Experimental

### 

#### Crystal data

C_14_H_10_BrClN_2_O_3_
*M* *_r_* = 369.60
Monoclinic, 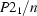

*a* = 8.279 (2) Å
*b* = 11.446 (3) Å
*c* = 14.998 (4) Å
β = 99.002 (4)°
*V* = 1403.7 (6) Å^3^
*Z* = 4
Mo *K*α radiation
μ = 3.13 mm^−1^
*T* = 298 (2) K
0.17 × 0.15 × 0.15 mm

#### Data collection

Bruker SMART CCD area-detector diffractometer
Absorption correction: multi-scan (*SADABS*; Sheldrick, 1996 ▶) *T*_min_ = 0.618, *T*_max_ = 0.651
10685 measured reflections
3006 independent reflections
2102 reflections with *I* > 2σ(*I*)
*R*_int_ = 0.049

#### Refinement

*R*[*F* ^2^ > 2σ(*F* ^2^)] = 0.039
*wR*(*F* ^2^) = 0.100
*S* = 1.03
3006 reflections
195 parameters
1 restraint
H atoms treated by a mixture of independent and constrained refinement
Δρ_max_ = 0.50 e Å^−3^
Δρ_min_ = −0.36 e Å^−3^

### 

Data collection: *SMART* (Bruker, 1998 ▶); cell refinement: *SAINT* (Bruker, 1998 ▶); data reduction: *SAINT*; program(s) used to solve structure: *SHELXS97* (Sheldrick, 2008 ▶); program(s) used to refine structure: *SHELXL97* (Sheldrick, 2008 ▶); molecular graphics: *SHELXTL* (Sheldrick, 2008 ▶); software used to prepare material for publication: *SHELXTL*.

## Supplementary Material

Crystal structure: contains datablocks global, I. DOI: 10.1107/S160053680802905X/bx2178sup1.cif

Structure factors: contains datablocks I. DOI: 10.1107/S160053680802905X/bx2178Isup2.hkl

Additional supplementary materials:  crystallographic information; 3D view; checkCIF report

## Figures and Tables

**Table 1:** Hydrogen-bond geometry (Å, °)

*D*—H⋯*A*	*D*—H	H⋯*A*	*D*⋯*A*	*D*—H⋯*A*
N2—H2⋯O3^i^	0.90 (3)	2.17 (3)	2.922 (3)	140 (3)
O1—H1⋯N1	0.82	1.91	2.623 (3)	146

Symmetry code: (i) 
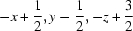
.

**Fig. 1.**
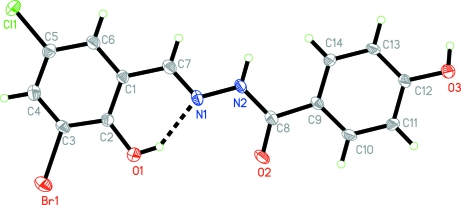
The structure of (I) with 30% probability displacement ellipsoids. dashed lines indicate intramolecular hydrogen bonds.

**Fig. 2.**
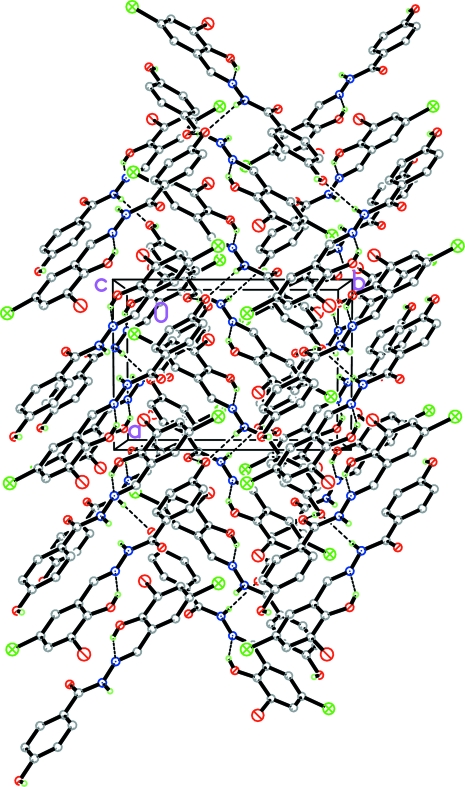
The molecular packing of (I). Intermolecular hydrogen bonds are shown as dashed lines. H atoms unrelated to the hydrogen bonding have been omitted for clarity.

## supplementary crystallographic information

### Comment

Schiff bases are a kind of versatile compounds, which possess excellent biological properties (Bhandari *et al.*, 2008; Sinha *et al.*, 2008; Sondhi *et al.*, 2006; Singh *et al.*, 2006). Recently, a large number of Schiff bases derived from the reaction of aldehydes with benzohydrazides have been reported (He & Liu, 2005; Zhen & Han, 2005; Diao & Yu, 2006; Shan *et al.*, 2008; Fun *et al.*, 2008). In this paper, a new Schiff base, (I), Fig. 1, derived from the reaction of 3-bromo-5-chlorosalicylaldehyde with 4-hydroxybenzohydrazide is reported. The title Schiff base is a planar-shaped compound, with mean deviation from the least-squares plane of 0.064 (3)Å, and with the dihedral angle between the C1-C6 and C9-C14 phenyl rings of 3.0 (2)°. All the bond lengths are comparable to the values in the similar Schiff bases (Jing *et al.*, 2005; Lu *et al.*, 2008; Salhin *et al.*, 2007). There is a intramolecular O—H···N hydrogen bond (Fig. 1). In the crystal structure, molecules are linked through intermolecular N—H···O hydrogen bonds , to form a 3-D network (Table 1) (Fig. 2).

### Experimental

3-Bromo-5-chlorosalicylaldehyde and 4-hydroxybenzohydrazide of AR grade were purchased from Aldrich and were used as was obtained. 3-Bromo-5-chlorosalicylaldehyde (235.3 mg, 1.0 mmol) and 4-hydroxybenzohydrazide (152.2 mg, 1.0 mmol) were dissolved in a methanol solution (80 ml). The mixture was stirred for two hours at room temperature. The resulting solution was left in air for a few days, yielding colourless block-like crystals.

### Refinement

H2 was located in a difference Fourier map and refined isotropically, with N—H distance restrained to 0.90 (3)Å, and with *U*_iso_(H) fixed at 0.08Å^2^. Other H atoms were placed in idealized positions and constrained to ride on their parent atoms with C—H distances of 0.93Å, O—H distance of 0.82Å, and with *U*_iso_(H) set at 1.2*U*_eq_(C) and 1.5*U*_eq_(O).

### Crystal data

**Table:**

C_14_H_10_BrClN_2_O_3_	*F*(000) = 736
*M**_r_* = 369.60	*D*_x_ = 1.749 Mg m^−^^3^
Monoclinic, *P*2_1_/*n*	Mo *K*α radiation, λ = 0.71073 Å
*a* = 8.279 (2) Å	Cell parameters from 2405 reflections
*b* = 11.446 (3) Å	θ = 2.3–24.6°
*c* = 14.998 (4) Å	µ = 3.13 mm^−^^1^
β = 99.002 (4)°	*T* = 298 K
*V* = 1403.7 (6) Å^3^	Block, colourless
*Z* = 4	0.17 × 0.15 × 0.15 mm

### Data collection

**Table:**

Bruker SMART CCD area-detector diffractometer	3006 independent reflections
Radiation source: fine-focus sealed tube	2102 reflections with *I* > 2σ(*I*)
graphite	*R*_int_ = 0.049
ω scans	θ_max_ = 27.0°, θ_min_ = 2.3°
Absorption correction: multi-scan (SADABS; Sheldrick, 1996)	*h* = −10→10
*T*_min_ = 0.618, *T*_max_ = 0.651	*k* = −14→14
10685 measured reflections	*l* = −19→19

### Refinement

**Table:**

Refinement on *F*^2^	Primary atom site location: structure-invariant direct methods
Least-squares matrix: full	Secondary atom site location: difference Fourier map
*R*[*F*^2^ > 2σ(*F*^2^)] = 0.039	Hydrogen site location: inferred from neighbouring sites
*wR*(*F*^2^) = 0.100	H atoms treated by a mixture of independent and constrained refinement
*S* = 1.03	*w* = 1/[σ^2^(*F*_o_^2^) + (0.0455*P*)^2^ + 0.1022*P*] where *P* = (*F*_o_^2^ + 2*F*_c_^2^)/3
3006 reflections	(Δ/σ)_max_ = 0.001
195 parameters	Δρ_max_ = 0.50 e Å^−^^3^
1 restraint	Δρ_min_ = −0.36 e Å^−^^3^

### Special details

**Table:**

Geometry. All esds (except the esd in the dihedral angle between two l.s. planes) are estimated using the full covariance matrix. The cell esds are taken into account individually in the estimation of esds in distances, angles and torsion angles; correlations between esds in cell parameters are only used when they are defined by crystal symmetry. An approximate (isotropic) treatment of cell esds is used for estimating esds involving l.s. planes.
Refinement. Refinement of F^2^ against ALL reflections. The weighted R-factor wR and goodness of fit S are based on F^2^, conventional R-factors R are based on F, with F set to zero for negative F^2^. The threshold expression of F^2^ > 2sigma(F^2^) is used only for calculating R-factors(gt) etc. and is not relevant to the choice of reflections for refinement. R-factors based on F^2^ are statistically about twice as large as those based on F, and R- factors based on ALL data will be even larger.

### Fractional atomic coordinates and isotropic or equivalent isotropic displacement parameters (Å^2^)

**Table:**

	*x*	*y*	*z*	*U*_iso_*/*U*_eq_	
Br1	1.16504 (5)	0.88843 (3)	1.28591 (2)	0.05440 (17)	
Cl1	1.19663 (10)	0.56024 (7)	1.02050 (6)	0.0436 (2)	
N1	0.7282 (3)	0.9996 (2)	0.98662 (16)	0.0299 (6)	
N2	0.6142 (3)	1.0530 (2)	0.92314 (15)	0.0303 (6)	
O1	0.9168 (3)	0.98960 (19)	1.14418 (14)	0.0409 (5)	
H1	0.8479	1.0183	1.1051	0.061*	
O2	0.5803 (3)	1.20014 (17)	1.01833 (13)	0.0387 (5)	
O3	0.0964 (3)	1.38132 (18)	0.68220 (14)	0.0426 (6)	
H3	0.0978	1.3553	0.6314	0.064*	
C1	0.9178 (3)	0.8455 (2)	1.02657 (18)	0.0275 (6)	
C2	0.9719 (3)	0.8893 (2)	1.11358 (19)	0.0278 (6)	
C3	1.0902 (4)	0.8268 (3)	1.17019 (18)	0.0314 (7)	
C4	1.1560 (4)	0.7256 (3)	1.1431 (2)	0.0351 (7)	
H4	1.2341	0.6847	1.1824	0.042*	
C5	1.1051 (4)	0.6856 (3)	1.0573 (2)	0.0323 (7)	
C6	0.9868 (4)	0.7438 (3)	0.9991 (2)	0.0324 (7)	
H6	0.9532	0.7149	0.9412	0.039*	
C7	0.7930 (4)	0.9061 (3)	0.9632 (2)	0.0327 (7)	
H7	0.7610	0.8760	0.9056	0.039*	
C8	0.5482 (4)	1.1559 (3)	0.94328 (19)	0.0283 (7)	
C9	0.4350 (3)	1.2130 (2)	0.87016 (17)	0.0258 (6)	
C10	0.3617 (4)	1.3161 (3)	0.89023 (19)	0.0344 (7)	
H10	0.3888	1.3482	0.9476	0.041*	
C11	0.2498 (4)	1.3723 (3)	0.8275 (2)	0.0375 (8)	
H11	0.2009	1.4412	0.8426	0.045*	
C12	0.2103 (4)	1.3258 (3)	0.74178 (18)	0.0287 (7)	
C13	0.2856 (4)	1.2251 (3)	0.71958 (18)	0.0349 (7)	
H13	0.2615	1.1948	0.6615	0.042*	
C14	0.3965 (4)	1.1692 (3)	0.78333 (19)	0.0339 (7)	
H14	0.4465	1.1009	0.7679	0.041*	
H2	0.577 (5)	1.016 (3)	0.8710 (15)	0.080*	

### Atomic displacement parameters (Å^2^)

**Table:**

	*U*^11^	*U*^22^	*U*^33^	*U*^12^	*U*^13^	*U*^23^
Br1	0.0585 (3)	0.0637 (3)	0.0345 (2)	0.00635 (19)	−0.01299 (16)	−0.01098 (17)
Cl1	0.0499 (5)	0.0294 (4)	0.0502 (5)	0.0096 (4)	0.0043 (4)	−0.0043 (4)
N1	0.0290 (13)	0.0281 (14)	0.0296 (13)	−0.0019 (11)	−0.0045 (10)	0.0072 (11)
N2	0.0334 (14)	0.0286 (14)	0.0251 (13)	0.0009 (11)	−0.0071 (10)	0.0026 (11)
O1	0.0468 (14)	0.0366 (13)	0.0358 (13)	0.0113 (11)	−0.0046 (10)	−0.0055 (10)
O2	0.0562 (14)	0.0317 (12)	0.0229 (11)	−0.0037 (10)	−0.0105 (9)	0.0004 (9)
O3	0.0609 (15)	0.0360 (13)	0.0264 (11)	0.0180 (11)	−0.0070 (11)	0.0015 (9)
C1	0.0285 (16)	0.0244 (16)	0.0278 (15)	−0.0028 (13)	−0.0009 (12)	0.0029 (12)
C2	0.0281 (16)	0.0255 (16)	0.0288 (15)	−0.0008 (13)	0.0006 (12)	−0.0007 (12)
C3	0.0298 (16)	0.0368 (18)	0.0251 (15)	−0.0048 (14)	−0.0040 (12)	−0.0032 (13)
C4	0.0330 (17)	0.0341 (18)	0.0368 (18)	0.0013 (14)	0.0011 (13)	0.0044 (14)
C5	0.0335 (17)	0.0258 (17)	0.0376 (17)	−0.0019 (13)	0.0056 (13)	−0.0002 (13)
C6	0.0358 (17)	0.0311 (18)	0.0283 (16)	−0.0039 (14)	−0.0009 (13)	0.0006 (13)
C7	0.0356 (18)	0.0313 (18)	0.0288 (16)	−0.0059 (14)	−0.0023 (13)	0.0023 (13)
C8	0.0301 (16)	0.0251 (16)	0.0274 (16)	−0.0075 (13)	−0.0023 (12)	0.0043 (12)
C9	0.0300 (16)	0.0243 (16)	0.0214 (14)	−0.0063 (13)	−0.0009 (11)	0.0043 (12)
C10	0.052 (2)	0.0295 (18)	0.0191 (14)	0.0013 (15)	−0.0020 (13)	−0.0031 (12)
C11	0.056 (2)	0.0253 (17)	0.0296 (17)	0.0130 (15)	−0.0001 (15)	−0.0038 (13)
C12	0.0373 (17)	0.0239 (16)	0.0227 (15)	0.0020 (13)	−0.0023 (12)	0.0057 (12)
C13	0.051 (2)	0.0319 (18)	0.0185 (15)	0.0053 (15)	−0.0046 (13)	−0.0041 (12)
C14	0.0457 (19)	0.0232 (16)	0.0299 (16)	0.0090 (14)	−0.0031 (13)	−0.0029 (13)

### Geometric parameters (Å, °)

**Table:**

Br1—C3	1.886 (3)	C4—C5	1.369 (4)
Cl1—C5	1.751 (3)	C4—H4	0.9300
N1—C7	1.271 (4)	C5—C6	1.378 (4)
N1—N2	1.375 (3)	C6—H6	0.9300
N2—C8	1.353 (4)	C7—H7	0.9300
N2—H2	0.90 (3)	C8—C9	1.478 (4)
O1—C2	1.343 (3)	C9—C10	1.381 (4)
O1—H1	0.8200	C9—C14	1.385 (4)
O2—C8	1.225 (3)	C10—C11	1.372 (4)
O3—C12	1.353 (3)	C10—H10	0.9300
O3—H3	0.8200	C11—C12	1.383 (4)
C1—C6	1.387 (4)	C11—H11	0.9300
C1—C2	1.404 (4)	C12—C13	1.376 (4)
C1—C7	1.464 (4)	C13—C14	1.376 (4)
C2—C3	1.390 (4)	C13—H13	0.9300
C3—C4	1.368 (4)	C14—H14	0.9300
			
C7—N1—N2	117.2 (2)	N1—C7—C1	120.4 (3)
C8—N2—N1	119.4 (2)	N1—C7—H7	119.8
C8—N2—H2	120 (3)	C1—C7—H7	119.8
N1—N2—H2	120 (3)	O2—C8—N2	121.8 (3)
C2—O1—H1	109.5	O2—C8—C9	121.4 (3)
C12—O3—H3	109.5	N2—C8—C9	116.8 (2)
C6—C1—C2	119.3 (3)	C10—C9—C14	118.0 (3)
C6—C1—C7	119.1 (3)	C10—C9—C8	117.7 (2)
C2—C1—C7	121.6 (3)	C14—C9—C8	124.3 (3)
O1—C2—C3	118.4 (3)	C11—C10—C9	121.6 (3)
O1—C2—C1	123.2 (3)	C11—C10—H10	119.2
C3—C2—C1	118.4 (3)	C9—C10—H10	119.2
C4—C3—C2	122.0 (3)	C10—C11—C12	119.5 (3)
C4—C3—Br1	120.1 (2)	C10—C11—H11	120.2
C2—C3—Br1	117.9 (2)	C12—C11—H11	120.2
C3—C4—C5	119.0 (3)	O3—C12—C13	122.0 (3)
C3—C4—H4	120.5	O3—C12—C11	118.2 (3)
C5—C4—H4	120.5	C13—C12—C11	119.8 (3)
C4—C5—C6	121.2 (3)	C14—C13—C12	120.0 (3)
C4—C5—Cl1	119.1 (2)	C14—C13—H13	120.0
C6—C5—Cl1	119.7 (2)	C12—C13—H13	120.0
C5—C6—C1	120.2 (3)	C13—C14—C9	121.0 (3)
C5—C6—H6	119.9	C13—C14—H14	119.5
C1—C6—H6	119.9	C9—C14—H14	119.5

### Hydrogen-bond geometry (Å, °)

**Table:**

*D*—H···*A*	*D*—H	H···*A*	*D*···*A*	*D*—H···*A*
N2—H2···O3^i^	0.90 (3)	2.17 (3)	2.922 (3)	140 (3)
O1—H1···N1	0.82	1.91	2.623 (3)	146.

Symmetry codes: (i) −*x*+1/2, *y*−1/2, −*z*+3/2.
